# Abnormal Connectivity and Brain Structure in Patients With Visual Snow

**DOI:** 10.3389/fnhum.2020.582031

**Published:** 2020-11-19

**Authors:** Njoud Aldusary, Ghislaine L. Traber, Patrick Freund, Fabienne C. Fierz, Konrad P. Weber, Arwa Baeshen, Jamaan Alghamdi, Bujar Saliju, Shila Pazahr, Reza Mazloum, Fahad Alshehri, Klara Landau, Spyros Kollias, Marco Piccirelli, Lars Michels

**Affiliations:** ^1^Department of Neuroradiology, Clinical Neuroscience Center, University Hospital Zurich, Zurich, Switzerland; ^2^Department of Diagnostic Radiology, Faculty of Applied Medical Sciences, King Abdulaziz University, Jeddah, Saudi Arabia; ^3^Department of Ophthalmology, University Hospital Zurich, University of Zurich, Zurich, Switzerland; ^4^Department of Ophthalmology, University of Basel, Basel, Switzerland; ^5^Institute of Molecular and Clinical Ophthalmology Basel, Basel, Switzerland; ^6^Spinal Cord Injury Center Balgrist, University of Zurich, Zurich, Switzerland; ^7^Department of Neurophysics, Max Planck Institute for Human Cognitive and Brain Sciences, Leipzig, Germany; ^8^Department of Neurology, University Hospital Zurich, University of Zurich, Zurich, Switzerland; ^9^Department of Radiological Sciences, Faculty of Applied Medical Sciences, King Saud University, Riyadh, Saudi Arabia; ^10^Institute of Robotics and Intelligent Systems, D-HEST, ETH Zurich, Zurich, Switzerland; ^11^Radiology, College of Medicine, Qassim University, Al Qassim, Saudi Arabia

**Keywords:** visual system, functional connectivity, visual snow, lingual gyrus, temporal cortex

## Abstract

**Objective:**

Visual snow (VS) is a distressing, life-impacting condition with persistent visual phenomena. VS patients show cerebral hypermetabolism within the visual cortex, resulting in altered neuronal excitability. We hypothesized to see disease-dependent alterations in functional connectivity and gray matter volume (GMV) in regions associated with visual perception.

**Methods:**

Nineteen patients with VS and 16 sex- and age-matched controls were recruited. Functional magnetic resonance imaging (fMRI) was applied to examine resting-state functional connectivity (rsFC). Volume changes were assessed by means of voxel-based morphometry (VBM). Finally, we assessed associations between MRI indices and clinical parameters.

**Results:**

Patients with VS showed hyperconnectivity between extrastriate visual and inferior temporal brain regions and also between prefrontal and parietal (angular cortex) brain regions (*p* < 0.05, corrected for age and migraine occurrence). In addition, patients showed increased GMV in the right lingual gyrus (*p* < 0.05 corrected). Symptom duration positively correlated with GMV in both lingual gyri (*p* < 0.01 corrected).

**Conclusion:**

This study found VS to be associated with both functional and structural changes in the early and higher visual cortex, as well as the temporal cortex. These brain regions are involved in visual processing, memory, spatial attention, and cognitive control. We conclude that VS is not just confined to the visual system and that both functional and structural changes arise in VS patients, be it as an epiphenomenon or a direct contributor to the pathomechanism of VS. These *in vivo* neuroimaging biomarkers may hold potential as objective outcome measures of this so far purely subjective condition.

## Introduction

Visual snow is a visual disturbance occurring in individuals with normal ophthalmic findings. It usually affects young adults and is characterized by the continuous perception of innumerable flickering dots (Schankin et al., 2014a). Interestingly, VS patients often draw the analogy to the flickering noise of a badly tuned analog television, even without previous knowledge about their condition. When associated with additional visual symptoms, it is referred to as “VS syndrome” (Schankin et al., 2014a; Puledda et al., 2018). Briefly, the latter involves VS as the main symptom with at least two additional visual symptoms from the following four categories: palinopsia, enhanced entoptic phenomena, photophobia, and nyctalopia. Tinnitus and migraine are the most commonly associated comorbidities of VS. Regarding migraine with or without aura (Schankin et al., 2014a,b), there is a suspected overlap in disease mechanism (Puledda et al., 2018), which represents one of the major challenges in VS research.

Until now, brain imaging studies showed neither any specific structural abnormalities (Bessero and Plant, 2014; Schankin et al., 2014a; Lauschke et al., 2016) nor any regional functional changes in cerebral water diffusion and perfusion (Jager et al., 2005). The latter study by Jager et al. (2005), however, only involved two patients with VS. Schankin et al. (2014b) investigated 17 patients with VS using FDG-PET and found hypermetabolism of the right lingual gyrus and left cerebellar anterior lobe (Schankin et al., 2014b). This finding is consistent with a disorder allocated downstream of the primary visual cortex, resulting in abnormal processing of visual information. In a few studies, occipital bending has been mentioned to co-occur in some patients with VS (Unal-Cevik and Yildiz, 2015; Yildiz et al., 2019) and may be related to depression (Maller et al., 2014, 2015). Recently, Puledda et al. (2020) reported metabolic and functional alterations using MR spectroscopy and task-based fMRI, respectively. The authors concluded that patients with VS demonstrated disturbed processing in the salience network, as the bilateral insular cortex showed lower BOLD signal responses in patients than those in controls. On the other hand, the elevated lactate concentration of the (right) lingual gyrus was interpreted as a sign of hyperexcitability in VS patients (Puledda et al., 2020).

Referring to the accepted notion that VS results from disturbed visual processing, this study investigates changes in resting-state fMRI of patients with VS compared to HCs within but also outside the visual cortex. We hypothesize to see alterations on a functional connectivity level as well as structural abnormalities within the visual system as a manifestation of the disease.

## Materials and Methods

### Participants and Clinical Data

Inclusion criteria: 19 patients over 18 years of age and meeting the diagnostic criteria for VS syndrome (Schankin et al., 2014a,b) were recruited consecutively at the Department of Ophthalmology, University Hospital Zurich, Switzerland. Exclusion criteria for all participants were pregnancy, presence of a neurodegenerative disorder, and contraindication against an MRI examination. The patients were all assessed by trained neuro-ophthalmologists and senior neurologists. Patients were age and sex matched to 16 HCs. In both patients and HCs, the history was completed with regard to symptoms and conditions associated with VS syndrome as shown in Table 1. The following clinical measures were included: duration of VS symptoms, history of migraine, tinnitus, anxiety, depression, tremor or imbalance, and perception of palinopsia, blue field entopic phenomena, other entoptic phenomena, photophobia, glare, nyctalopia, symptoms in darkness, symptom presence with eyes closed, and overall perceived symptom severity on a scale of 0–10. Migraine occurrence was assessed with the Diagnostic Algorithm of the Hardship Questionnaire (Steiner et al., 2014). For one HC, we could not receive any feedback on the migraine status and thus modeled migraine presence with “0.5” in both types of analysis. None of the VS patients showed any signs of an underlying ophthalmic pathology based on the history and the clinical examination including best corrected visual acuity, static perimetry (Octopus 900, Haag-Streit, Bern, Switzerland), fundoscopy, and optical coherence tomography of the macula and the peripapillary retinal nerve fiber layer (Heidelberg Spectralis, Heidelberg Engineering, Heidelberg, Germany). All subjects gave informed written consent to participate in this study, which was approved by the ethics committee, Canton Zurich, Switzerland (BASEC-NR: 2016-00225).

**TABLE 1:** Summary of demographic and clinical values for visual snow (VS) patients and healthy controls (HCs).

				Non-visual symptoms							Visual symptoms							Cortical changes	
ID	Age (years)	Sex	Duration of VS years)	Migraine	With aura	Tinnitus	Anxiety	Depression	Tremor	Imbalance	Palinopsia	Blue field entoptic phenomenon	Other entoptic phenomena	Photophobia	Glare	Nyctalopia	Symptoms in darkness and with eyes closed	Volume increase	Occipital bending
VS1	28	M	3	−	−	+	−	−	+	−	−	−	+	+	+	−	+	Left occipital	−
VS2	44	F	9	+	−	+	−	+	−	+	−	−	+	−	−	+	+	−	−
VS3	47	M	17	+	+	+	−	+	−	+	−	+	−	−	+	+	+	Left occipital	Left occipital
VS4	23	M	6	−	−	−	−	+	−	−	−	+	−	−	−	+	+	−	−
VS5	33	M	4	−	−	+	+	−	−	−	−	−	+	+	+	−	+	−	−
VS6	18	M	1	−	−	+	+	+	+	+	+	+	−	+	−	+	+	−	−
VS7	19	M	19	−	−	−	−	−	+	−	−	−	+	−	−	−	+	−	−
VS8	44	F	4	+	+	+	+	+	−	−	+	−	−	+	+	+	+	−	−
VS9	30	F	5	+	+	+	−	−	−	−	+	−	−	+	−	−	+	Left occipital	−
VS10	39	F	1	+	+	+	+	−	−	−	−	+	−	+	+	+	+	−	−
VS11	58	M	38	+	+	+	+	−	+	−	−	+	+	+	+	+	+	+	Left occipital
VS12	33	M	2	−	−	−	−	−	−	−	−	+	+	+	−	−	+	−	Right occipital
VS13	21	M	1	+	+	+	−	−	−	+	+	−	+	−	+	−	−	−	−
VS14	54	F	0.5	+	−	−	−	+	−	−	+	+	+	+	−	−	+	−	Left occipital
VS15	21	F	4	+	+	−	+	+	−	−	−	+	+	−	−	−	+	+	Left occipital
VS16	38	M	3	+	+	−	−	−	−	−	−	+	+	+	−	−	+	−	Right occipital
VS17	30	F	0.5	−	−	+	−	−	−	−	−	+	+	−	−	−	+	+	Left occipital
VS18	22	M	> 5	−	−	+	−	−	−	−	−	−	−	−	+	+	+	−	−
VS19	30	M	5	−	−	−	−	−	−	−	−	+	+	−	−	+	+	−	−
HC1	31	F	−	+	−	−	−	−	−	−	−	−	−	−	−	−	−	−	−
HC2	37	M	−	−	−	−	−	−	−	−	−	−	−	−	−	−	−	−	−
HC3	32	M	−	−	−	−	−	−	−	−	−	−	−	−	−	−	−	−	−
HC4	29	M	−	n/a	n/a	−	−	−	−	−	−	−	−	−	−	−	−	−	−
HC5	31	F	−	−	−	−	−	−	−	−	−	−	+	−	−	−	−	−	−
HC6	28	M	−	−	−	−	−	−	−	−	−	−	−	−	−	−	−	−	−
HC7	21	M	−	−	−	−	−	−	−	−	−	−	+	−	−	−	−	−	−
HC8	40	M	−	+	−	−	−	−	−	−	−	−	−	−	−	−	−	−	−
HC9	49	F	−	+	−	−	−	−	−	−	−	−	−	−	−	−	−	−	−
HC10	33	F	−	+	+	−	+	+	−	−	−	−	−	−	−	−	−	−	−
HC11	37	M	−	−	−	−	−	−	−	−	−	−	−	−	−	−	−	−	−
HC12	18	F	−	−	−	−	−	−	−	−	−	−	−	−	−	−	−	−	−
HC13	29	F	−	−	−	−	−	−	−	−	−	−	−	−	−	−	−	−	−
HC14	24	F	−	−	−	−	−	−	−	−	−	−	−	−	−	−	−	−	−
HC15	34	M	−	−	−	−	−	−	−	−	−	−	−	−	−	−	−	−	−
HC16	32	F	−	−	−	−	−	−	−	−	−	−	−	−	−	−	−	−	−

### Statistics: Demographics

Independent two-sample *t*-tests or chi-square tests were performed to test for (group) differences in age and sex. The Shapiro–Wilk test was used to evaluate normal distribution of demographic variables.

### MRI

All participants were scanned on a 3-T MRI Scanner (Philips Healthcare, Best, Netherlands) with a two-channel transmit and 32-channel receive phase-array head coil. A three-dimensional-encoded T1-weighted (T1w) Turbo Field Echo inversion recovery sequence was acquired with the following parameters: field of view (FOV): 240 × 197 × 170 mm^3^; acquired voxel size: 0.9 × 0.9 × 0.9 mm^3^; SENSE factors 2 × 2; slice orientation: transverse; turbo field factor: 104; profile order: linear; flip angle: 8°; water fat shift: 2.5 pixels; inversion time: 950 ms; TR/TE: 9.1/4.2 ms; TFE shot interval: 1,434 ms; acquisition time: 3.2 min.

### Resting-State Functional MRI

Functional images were acquired with an EPI with the following parameters: TR: 2,000 ms; TE: 35 ms; FA: 79°; FOV: 210 mm^2^; 76 × 76 matrix; ascending slice order; voxel resolution: 2.76 × 2.84; in-plane thickness: 2.90 mm; 10:13 min scan time. All participants were instructed to keep their eyes closed during the scan. To minimize head motion, comfortable pillows were placed around the participant’s head.

### Functional Analysis

Pre-processing and analysis of the resting-state fMRI data were done using the “CONN” toolbox (Version 17f)^1^ (Whitfield-Gabrieli and Nieto-Castanon, 2012). Pre-processing included standard steps for fMRI, i.e., slice time correction, realignment and adjustment for movement-related effects, functional outlier detection (scrubbing) based on ART, normalization of the functional data to the standard stereotactic MNI space, smoothing with an isotropic Gaussian kernel of 6 mm FWHM. CONN accounts for bad data points (using the “ART detection” toolbox) by including bad data point and movement time courses as nuisance regressors during the denoising procedure. Thus, data are not being inserted or interpolated with CONN. The estimate of head motion differences between groups was performed using the CONN toolbox calculator by computing the average displacement on x, y, and z dimension for each participant, and then we calculated the differences in translation between groups. The residual BOLD time series was bandpass filtered between 0.01 and 0.1 Hz to reduce the effect of slow frequency drifts and high-frequency noise. Only the WM and CSF signals were removed to avoid any bias introduced by removing the global signal [i.e., GM]. This denoising step has been shown to “normalize” the distribution of voxel-to-voxel connectivity values as effectively as including the global signal as a covariate of no interest but without the potential problems of the latter method (Behzadi et al., 2007; Murphy et al., 2009). Additionally, linear detrending was performed during the denoising step. After denoising, the distribution of voxel-to-voxel connectivity was visualized for each step. All participants showed normally distributed data ensuring high data quality. Displacement values, time courses of scrubbed data points, white matter volume (WMV) signal, CSF signal, and global signal intensity were used as covariates of no interest for the subsequent statistical analyses.

### Statistics: Region of Interest Resting-State Functional Connectivity Analysis

To examine rsFC between HC and VS patients, we performed ROI-to-ROI analyses across non-primary visual cortical and subcortical regions (from the FSL Harvard Oxford atlas) and *a priori* anatomically defined ROIs of the visual cortex (in total 167 ROIs). These regions are imported from the SPM12 anatomy toolbox Version: 2.2c (Eickhoff et al., 2005) and are based on probabilistic cytoarchitectonic maps (i.e., the cytoarchitectonic maps’ superimposition with fMRI data). This approach has been shown to provide a valuable method to assess the group functional activity of striate and extrastriate visual areas (Wilms et al., 2010). These ROIs subdivide the visual cortex in each hemisphere into several regions, namely, primary and secondary visual cortex (V1 and V2, respectively), ventral extrastriate cortex (hOc3V, V3v), hOc4v (V4), dorsal extrastriate cortex (hOc5, V5/MT+), hOc3d (V3d), hOc4d (V3A), and posterior fusiform gyrus (FG1 and FG2). We did not include the cerebellum as only part of the cerebellum was covered. In particular, Pearson correlations were calculated between time courses of the described ROIs. Fisher-transformed correlation maps (i.e., Fisher-transformed correlation coefficients) were used for second-level between-group analyses. For all analyses, significant results were only reported if they survived a connection threshold of *p* < 0.05 [using a false discovery rate (FDR) seed level correction] with an additional cluster-threshold of *p* < 0.05 (uncorrected). As a *post hoc* analysis, the main analysis was repeated using the seed of the right lingual gyrus based on our structural findings. We also recomputed the analysis by removing the two controls with entoptic phenomena or with a previous episode of anxiety and depression.

### Structural Analysis

Voxel-based morphometry implemented in SPM^2^ was applied to T1w MPRAGE images to assess differences in GMV between patients and controls (Ashburner and Friston, 2000). We segmented T1w MPRAGE images into GM, WM, and CSF with unified segmentation (Ashburner and Friston, 2005). Next, the GM segments were spatially normalized into standard MNI space, with a diffeomorphic Anatomical Registration using Exponentiated Lie algebra (DARTEL) algorithm (Ashburner, 2007). The GMV of each voxel was obtained through modulation. Finally, the GMVs were scaled with the Jacobian determinants estimated by the registration step (i.e., “modulation”) in order to preserve the local tissue volumes and smoothed using an isotropic Gaussian kernel with 6 mm FWHM.

### Statistics: Structural Analysis

Cluster inference was performed using a cluster-defining threshold of *p* = 0.001 and an FWE corrected threshold of *p* = 0.05 using Gaussian random field theory to account for multiple comparisons (Friston et al., 1994) within predefined ROIs based on *a priori* hypothesis. Only significant results (*p* < 0.05) corrected for FWE and adjusted for age, total intracranial volume, and migraine are reported. The ROIs included the bilateral lingual gyri (Schankin et al., 2014b), derived from the WFU PickAtlas^3^ (version 2.3) (Maldjian et al., 2003).

### Statistics: Correlation of Neuroimaging Measures to Clinical Parameters

Regression analysis was performed to examine the interaction of rsFC and regional GMV to symptom duration and symptom severity (range: 0–10; adjusted for age and migraine).

## Data Availability Statement

Anonymized data will be shared by request from any qualified investigator.

## Results

### Demographic and Clinical Characteristics of Participants

All demographic data were normally distributed (all *p* > 0.05, Shapiro–Wilk test). Nineteen patients with VS (mean age 33.3 ± 11.5 years, range: 18.3–58.0 years, 7/19 women) and 16 HCs (mean age 31.6 ± 7.3 years, range: 18.1–48.8 years, 8/16 women) participated in the study. The difference in age (*t* = 0.5, *p* = 0.62) and gender (*p* = 0.51, chi-square test) between the groups was not statistically significant. Fifteen patients and 14 HCs were right-handed. Demographic and clinical data of VS patients are summarized in Table 1. None of the HCs had any of the symptoms or associated conditions listed in Table 1. Mean duration of visual symptoms in the patients was 6.7 ± 11.1 years. Ten of 19 patients had comorbid migraine with eight of 10 having migraine with visual aura. Migraine was present in four of the HCs (one with visual aura).

### Functional MRI: Resting-State Functional Connectivity Analysis

**FIGURE 1:**
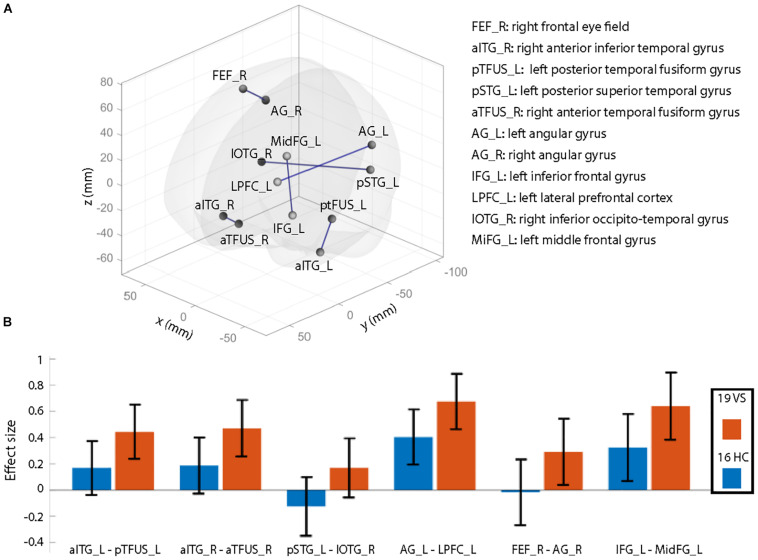
Illustration of resting-state functional connectivity (rsFC) differences comparing healthy controls (HCs) to patients with visual snow (VS). **(A)** Patients with VS showed significantly increased rsFC (connections labeled in blue) compared to that in HCs. Results are shown at *p* < 0.05 (all *t*-values > 3.3), false discovery rate (FDR) seed level corrected (with additional correction for age and migraine occurrence). X, Y, and Z denote Montreal Neurological Institute (MNI) coordinates. **(B)** Effect sizes for both groups for significant rsFC between-group differences (^∗^*p*-values). These plots are shown for illustrative purposes only to demonstrate that the observed effects are mainly due to higher positive correlations in patients than in controls (i.e., they are not the result of weaker anticorrelations between regions). Abbreviations: atFUS, anterior temporal fusiform gyrus; pTFUS, posterior temporal fusiform gyrus; IOTG, inferior occipito-temporal gyrus; AG, angular gyrus; IFG, inferior frontal gyrus; aITG, anterior inferior frontal gyrus; IFG, inferior frontal gyrus; MidFG, middle frontal gyrus; FEF, frontal eye field; pSTG, posterior superior temporal gyrus; LPFC, lateral prefrontal cortex; L, left; R, right.

Groups did not differ significantly in the number of movement artifacts related to the mean of each translation direction (all *p* > 0.1). Also, there was no group difference for maximal motion (largest motion observed, *p* = 0.37) or mean motion (*p* = 0.80). The number of removed frames during scrubbing was not significantly different between groups (*p* > 0.1) as well as the number of valid scans (*p* = 0.99). After controlling for age and migraine, rsFC differences—illustrated in Figure 1A—were seen as hyperconnectivity (VS > HC) for the following connections:

(1) Left anterior inferior temporal gyrus (aITG) – left posterior temporal fusiform gyrus (pTFUS)
(2) Right aITG – right anterior temporal fusiform gyrus (aTFUS)
(3) Left posterior superior temporal gyrus (pSTG) – right inferior occipito-temporal gyrus (IOTG)
(4) Left angular gyrus (AG) – left lateral prefrontal cortex (LPFC)
(5) Right frontal eye field (FEF) – right AG
(6) Left inferior frontal gyrus (IFG) – left middle frontal gyrus (MidFG).

The effect sizes for each group are visualized in Figure 1B. Positive correlations equal a positive effect size. Since most of the connections reveal positive effect sizes, the observed hyperconnectivity (Figure 1A) can be interpreted as higher positive correlations in the VS group compared to HC (and not as weaker anticorrelations). Results did not change when removing the three controls with either entoptic phenomena or a previous episode of anxiety and depression. As a *post hoc* analysis, we repeated the rsFC analysis but included the right lingual gyrus (see section “Structural Analysis”) as additional seed region. The results did not deviate from our original findings, i.e., no additional rsFC abnormality was seen from the lingual gyrus to other brain regions in patients with VS.

### Structural Analysis

**FIGURE 2:**
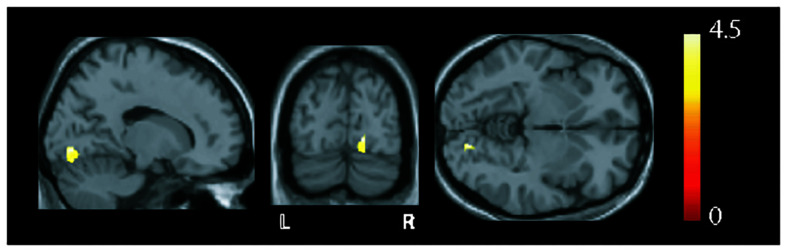
Overlay of structural differences (shown in sagittal, coronal, and axial slices) between healthy controls (HCs) and patients with visual snow (VS). Increases in gray matter volume were seen in the right (R) lingual gyrus [*p* = 0.014, family-wise error (FWE) corrected, *t*-value = 4.61] in the patient group when compared to controls.

MRI findings show (as judged by the neuroradiologist) occipital bending in seven patients with VS and in none of the HCs (Table 1). Based on the VBM analysis (Figure 2), higher GMV was found in the VS group in the right lingual gyrus (x = 17, y = −81, z = −5, z = 4.61, *p* = 0.014, FWE corrected).

### Correlation of Neuroimaging Measures to Clinical Parameters

**FIGURE 3:**
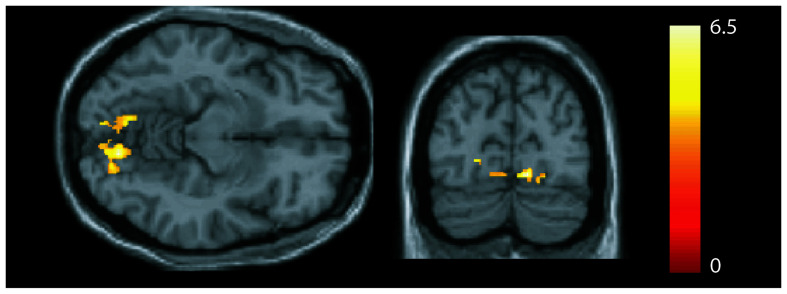
Association between gray matter volume and disease duration. A positive correlation was seen in the bilateral lingual gyrus [*p* < 0.05, family-wise error (FWE) corrected].

We observed a positive (Figure 3) correlation in patients between symptom duration and the GMV of both lingual gyri (MNI coordinates left: x = −8, y = −72, z = −11: *z* = 3.99, *p* = 0.002; MNI coordinates right: x = 12, y = −78, z = −11; statistics: *z* = 4.62, *p* < 0.001, FWE corrected). No correlations were seen between GMV (or FC strength) and symptom severity.

## Discussion

The main findings of this study were hyperconnectivity in the visual and prefrontal cortex and higher GMV in the right lingual gyrus in patients with VS compared to controls. This suggests that both functional and structural plastic changes are important hallmarks of the pathophysiology involved in VS.

### Functional Alterations

So far, disturbed processing of visual information downstream the primary visual cortex seems to be the most likely site of origin as outlined in a recent review article (Traber et al., 2020). Earlier work by Schankin et al. (2014b) reported hypermetabolism (using PET) of the lingual gyrus and cerebellar anterior lobe in 17 patients with VS, already indicating disease-related effects allocated downstream of the primary visual cortex. In our study, we applied resting-state fMRI, which allows to examine spontaneous low-frequency fluctuations of the BOLD signal in the absence of external tasks or stimuli. This MRI technique can provide a measure of rsFC between various brain regions (Fox and Raichle, 2007) and networks (Damoiseaux et al., 2006). It has been applied in various clinical populations including patients with migraine (Mainero et al., 2011; Schwedt et al., 2013; Yuan et al., 2013) or medication overuse headache (Michels et al., 2017). We did not find rsFC alterations in the lingual gyrus but in other areas of the frontal cortex (FEF), extrastriate visual cortex (pSTG), and temporal cortex (aITG, aTFUS, pTFUS, and IOTG). The extrastriate visual cortex is engaged in object recognition, spatial attention, and global motion and is interconnected with other structures essential for memory (Mishkin, 1982; Wicker et al., 1998; Rizzolatti and Matelli, 2003; Tong, 2003; Moriguchi et al., 2009). Our results thus indicate that VS leads to a disturbed interplay between various areas within the visual cortex. Recently, abnormal resting-state fMRI signal responses have also been noted in the FEF in patients with VS (Puledda et al., 2020). The FEF (but also the supramarginal gyrus) is involved not only in the control of eye movements but also in the control of visual awareness and visuospatial attention (Vernet et al., 2014; Quentin et al., 2015), and hyperconnectivity of this region to the AG might indicate abnormal attentional control. It has also been demonstrated that the STG showed decreased GMV as well as abnormal perfusion (Schankin et al., 2020). Recent evidence suggests that—among other regions—the STG might be associated with the development of tinnitus (Liu et al., 2018). In our study, the hyperconnectivity of this region might represent a pathophysiological correlate of tinnitus, as 12 out of 19 patients showed tinnitus. The observed hyperconnectivity of the AG to and within the prefrontal cortex (LPFC) suggests that VS impairs the network integrity of higher cognitive areas such as the IFG, LPFC, and contralateral AG. The hyperconnectivity of the IFG, LPFC, FEF, and IFG could index interference with cognitive control (Cieslik et al., 2013) or with visual search functionality in patients with VS (Schall, 2002; Nelson et al., 2016; Reteig et al., 2018). The AG is involved in higher cognitive function, such as calculation and symbol processing (Price and Ansari, 2011), but it also computes action awareness representations (Farrer et al., 2008). Using transcranial direct current stimulation over the AG leads to modulated priming of visual search (Taylor et al., 2011) and can disturb visuo-proprioceptive perception (Block et al., 2013). The latter is particularly interesting given that VS has also been suspected to be a disorder of heightened perception of normal sensory phenomena (Moster and Tariq Bhatti, 2019). Few patients in our study had palinopsia, which is defined as the inability to suppress the just-seen (Critchley, 1951). There is a case report demonstrating hypometabolism in the inferior parietal lobule, particularly in the AG (Hayashi et al., 2002). Thus, hyperconnectivity of the AG might be related to abnormal metabolism in this region, but to examine this in more detail, PET or arterial spin labeling imaging should be applied in patients with VS consistently showing palinopsia.

A recent study reported alterations in neurotransmission and fMRI signal strength during visual stimulation mimicking VS (Puledda et al., 2020). The authors observed reduced bilateral anterior insula BOLD responses to the visual stimulus with respect to baseline in VS patients compared to controls. In addition, an increase in lactate concentration was found in patients compared to controls in the right lingual gyrus. We did not find alterations in the rsFC of the insular cortex, which might be due to the lack of active visual stimulation leading to altered connectivity of the salience network. However, our rsFC data suggest that, in VS patients, visual processing is dysfunctional even in the absence of visual stimuli, which goes in line with the commonly reported worsening of symptoms in darkness or with eyes closed.

### Gray Matter Alterations

Strikingly, we found higher GMV in the lingual gyrus; its magnitude being associated with disease duration. Consistent with our finding, hypermetabolism of the lingual gyrus and cerebellar anterior lobe (by means of PET) was seen in 17 patients with VS (Schankin et al., 2014b). Our results and the latter finding of Schankin et al. (2014b) indicate that disease-related effects in this region are detectable at both the functional and the structural level. Remarkably, the locations of hypermetabolism and structural alterations found in the two studies almost overlap on normalized brain coordinates, although different imaging modalities were used (PET vs. T1-weigted volume analysis) and different cohorts of VS patients were examined. In addition, we found that a longer disease duration correlated with higher GMV of the right lingual gyrus. As hypothesized earlier (Kutch et al., 2017), increased GMV (which mainly reflects dendrites and axons) may reflect a dynamic strengthening in synaptic strength and synaptic plasticity processes (increased synaptic activity) as a result of dendritic branching or axonal sprouting. In case of VS, this neuroplastic effect may reflect chronicity seen as changes in a regional structural alteration.

## Conclusion

This multimodal imaging study found VS to be associated with regional structural and functional alterations in the early and higher visual cortex, as well as with hyperconnectivity to the temporal cortex. The involved brain regions are related to visual processing, memory, spatial attention, and cognitive control. Both functional and structural changes arise in VS patients, be it as an epiphenomenon or as a direct contributor to the pathomechanism of VS. The tight link of GMV in both lingual gyri to symptom duration underlines the critical role of the lingual gyrus in disease manifestation. These *in vivo* neuroimaging biomarkers may hold potential as objective measures of this intriguing condition, which cannot yet be objectively quantified.

## Data Availability Statement

The raw data supporting the conclusions of this article will be made available by the authors, without undue reservation.

## Ethics Statement

The studies involving human participants were reviewed and approved by Canton Zurich, Switzerland (BASEC-NR: 2016-00225). The patients/participants provided their written informed consent to participate in this study.

## Author Contributions

LM and PF analyzed the data and wrote the paper. GT was involved in the study design and wrote the manuscript. SK was involved in the study design and manuscript drafting. All other authors helped with data recording and data analysis.

## Conflict of Interest

The authors declare that the research was conducted in the absence of any commercial or financial relationships that could be construed as a potential conflict of interest.

## Footnotes

1 http://www.nitrc.org/projects/conn

2 http://www.fil.ion.ucl.ac.uk/spm

3 http://www.fmri.wfubmc.edu/cms/software

## Abbreviations

ART: artifact detection tools
BOLD: blood oxygen level-dependent
CSF: cerebrospinal fluid
EPI: echo-planar imaging
FDG-PET: [(18)F]-2-fluoro-2-deoxy-D-glucose positron emission tomography
fMRI: functional magnetic resonance imaging
FWE: family-wise error
FWHM: full width at half maximum
GM: gray matter
GMV: gray matter volume
HC: healthy control
MNI: Montreal Neurological Institute
MPRAGE: magnetization-prepared rapid gradient-echo
ROI: region of interest
rsFC: resting-state functional connectivity
SD: standard deviation
VBM: voxel-based morphometry
VS: visual snow
WM: white matter.
